# Comparative cardiac effects of antimalarial drug halofantrine with or without concomitant administration of kolanut or fluconazole in healthy volunteers

**DOI:** 10.4314/ahs.v23i1.28

**Published:** 2023-03

**Authors:** Aduragbenro DA Adedapo, Dike B Ojji, Kayode S Adedapo, Yetunde Kolade, Chinedum P Babalola

**Affiliations:** 1 Department of Pharmacology and Therapeutics, University of Ibadan, Nigeria; 2 Department of Clinical Pharmacology, University College Hospital, Ibadan, Oyo State, Nigeria; 3 Cardiology Unit, Medicine Department, Gwagwalada Hospital, Abuja, Nigeria; 4 Department of Chemical Pathology, University of Ibadan, Ibadan, Nigeria; 5 Department of Pharmaceutical Chemistry, University of Ibadan, Ibadan, Nigeria; 6 Reckitt Benckiser, Bath Road, Slough, Berkshire, SL 1 3 UH, United Kingdom

**Keywords:** Drug interaction, fluconazole, halofantrine, kolanut, malaria, QT prolongation

## Abstract

**Background:**

There is rekindled interest in the cardiotoxicity of antimalarial medicines. Halofantrine is associated with QT interval prolongation. Fluconazole and kolanut alter the pharmacokinetics of halofantrine.

**Objectives:**

The study assessed the electrocardiographic changes of concomitant administration of kolanut or fluconazole with halofantrine and the effects on the QTc interval.

**Methods:**

Eighteen healthy volunteers received a single oral dose of halofantrine, halofantrine with kolanut or halofantrine with fluconazole in a crossover study. Twelve lead electrocardiography (ECG) was performed to measure the PR and QT interval (QTc). Statistical analysis was with SPSS at 5% level of significance.

**Results:**

PR intervals were shortened by halofantrine alone and halofantrine with kolanut (169.29 28.67 to 165.29 28.007 and 172.73 29.843 to 163.00 18.336ms) but was prolonged by halofantrine with fluconazole (177.70 27.394 to 186.59 44.434ms). There was prolongation of QTc (384.76 21.727 to 394.12 21.525; 381.36 22.29 to 388.30 17.26 and 382.35 20.08 to 390.84 21.97) in all the three treatment groups at 6 hours, p>0.05. One subject on halofantrine and fluconazole had QTc >440ms. Pre-treatment PR interval (PR_0_) correlated well with post-treatment PR_6_, and with PR_14_ r= 0.519, p= 0.014; r=0.664, p=0.013.

**Conclusion:**

Concomitant intake of kolanut with halofantrine was significantly decrease cardiac effect of halofantrine.

## Introduction

Malaria is the most important parasitic infection affecting man and may coexist with other infections including HIV. Malaria is a close differential diagnosis of corona virus disease-19 (COVID-19) in this current pandemic. Halofantrine is a synthetic phenanthrene-methanol antimalarial which is chemically related to quinine and mefloquine. Although halofantrine, a potentially cardiotoxic drug is an efficacious antimalarial for the treatment of multi-drug resistant *P. falciparum* malaria, it never was one of the drugs recommended for the treatment of malaria by the World Health Organization^1^.^2^, it can cause rare but fatal cardiac complications^3^. Halofantrine has been associated with QT interval prolongation in patients without known cardiac abnormalities^4^,^5^. It is also associated with fatal and non-fatal arrhythmias in some person^6^,^7^. As a result of this, many experts recommend that halofantrine be used for treatment only in persons who have a normal electrocardiogram, which obviously makes its use in many less-developed settings impractical 4. However, several drugs including antimalarial drugs are being repurposed for the treatment of COVID-19, some contrary to WHO recommendation^8^ (Off label). Halofantrine treatment may be more dangerous in patients with cardiac abnormalities or those who are taking mefloquine for malaria prophylaxis. In both settings, Q-T prolongation may occur more frequently^6^. In the presence of Q-T interval prolongation, there is an increased risk of the development of Torsades de pointes, which is a potentially life-threatening arrhythmias^9^ and sudden death may occur. Other medications and conditions associated with prolonged Q-T interval include: quinine, sotalol, disopyramide, amiodarone, macrolides, antipsychotic agents, hypokalemia, bradycardia, congestive cardiac failure, subclinical long QT syndrome and severe hypomagnesemia^9^. Fluconazole an antifungal agent is an established part of therapy in HIV patients and thus may be co-administered with halofantrine in HIV patients having malaria. Kolanut commonly consumed by Africans contains caffeine, which may enhance the aqueous solubility of halofantrine has been reported to adsorb halofantrine thereby forming a complex^10^. In spite of the high prevalence of malaria and HIV in Nigeria, and the possible co-administration of halofantrine and fluconazole as well as patient consumption of kolanut, there has not been any full report of the cardiac effect of halofantrine when co-administered with fluconazole or kolanut. Effects of the co-administration on the plasma concentration of halofantrine and N-desbutylhalofantrine have been reported^10^,^11^. Halofantrine causes dose-related prolongation of QT and PR intervals^12^. Various factors influence QT changes, including physiological fluctuations related to postures, exercise, meals, and time of the day, which need to be differentiated from drug-induced QT changes^13^. Interest in the cardiotoxicity of antimalarial medicines is rekindled in view of the report of QTc prolongation with the currently recommended first line ACTs, dihydroartemisinin-piperaquine^2^. However, there are controversies, Gutman and colleagues reported QTc that was within normal limit in all 14,628 participants who were administered DHA-PPQ^14^, Bindschedler and colleagues reported QTc interval prolongation in all 13 participants following a single dose of 500mg halofantrine^15^. A call made for “thorough QT” assessment^2^ necessitates a detailed report of the effect of other commonly used drugs for common chronic infective and infectious disease on the cardiac effect on halofantrine which is highly needed but is unfortunately still lacking. This underscores the need for a full report previously published as an abstract^16^.

## Objectives

The study assessed the electrocardiographic changes of concomitant administration of kolanut or fluconazole with halofantrine to determine the effects on the QTc and PR intervals. The study also evaluated the influence of serum potassium on the electrocardiographic findings.

## Materials and methods

The study is part of a larger study conducted between September 2003 and January 2004 for which joint University of Ibadan/University College Hospital (UI/IRC/02/0041) ethical approval was obtained^10^,^11^. Eighteen healthy male volunteers, after informed consent were screened, enrolled and randomized to receive a single oral dose of halofantrine (500mg), halofantrine with kolanut (12.5mg) or halofantrine with fluconazole (25mg) at random order, during a crossover study, with a wash out period of 6 weeks between treatments. Minimum wash out period was two weeks, up to 5 weeks was allowed considering seven half-lives (4-7 half-lives, usually accepted for elimination) for caffeine, choice of six weeks was due to logistics. Background assessment and abnormalities were evaluated by a physician. Those with background abnormalities based on history, physical examination and electrocardiographic parameters were excluded from the study.

Clinical information including family history of heart disease was obtained. Physical examination included blood pressure measurement at baseline and at follow up. Twelve lead electrocardiography (ECG) was performed at 0, 6 and 336 hours with a portable ECG machine P80Six, Esaotebiomedica, Italy, to measure the PR and QT interval (QTc). Electrocardiographic parameters were automatically generated by the machine and confirmed by the authors. Heart rate (Hr.) beats/minutes was measured from the R-R interval by dividing the number of large squares (0.20 s) time unit between consecutive R waves into 300 or the small square (0.04 s) time units into 1500^17^. (Goldberger, 2012). P wave which denotes atrial depolarization was from the beginning to the end of the P wave. PR interval (normally 120-200 ms) which measures the interval between atrial and ventricular depolarization was measured from the beginning of P wave to the beginning of the QRS wave. QRS which denotes ventricular depolarization was measured from the beginning to the QRS wave to the J point^18^ (Hong et al., 2014). QT interval was measured manually, by using one of the limbs leads that best shows the end of the T wave on a 12-lead ECG. And the QT interval was measured from the beginning of the QRS complex to the end of the T wave and averaged over 3 to 5 beats. And this was then corrected using the Bazett formula, QTc = QT / √RR., this denotes ventricular contraction. Blood chemistry was performed on eight of the subjects, halofantrine group 2, halofantrine plus kolanut 4 and halofantrine plus fluconazole 2 subjects at baseline. This was due to financial constraints.

Statistical analysis was with SPSS at 5% level of significance.

## Results

The baseline clinico-electrocardiographic characteristics of healthy volunteers administered halofantrine alone or with either fluconazole or kolanut as shown in Table 1a, were not different in all the groups except for diastolic blood pressure (DBP) in halofantrine plus kolanut group.

**Table 1a:** Baseline clinico-electrocardiographic characteristics of healthy volunteers administered halofantrine alone or with either fluconazole or kolanut

Characteristics	All Subjects N = 48	Halofantrine n = 17	Halofantrine with kolanut n = 11	Halofantrine with fluconazole n = 20
Age (yr)	23.1 ± 2.3	22.7 ± 2.5	22.4 ± 2.5	23.9 ± 1.9
Systolic Blood Pressure (mmHg)	110.0 ± 12.9	112.5 (9.9)	111.0 (15.2)	106.7 (15.1)
Diastolic Blood Pressure	69.7 ± 10.2	64.2 (6.6)	79.0 (10.2) *	67.5 (8.8)
Heart Rate 0 hr	68.6 ± 11.5	68.8 ± 11.5	64.1 ± 10.5	70.9 ± 11.9
RR 0 hr	912.6 ± 142.8	908.7 ± 143.4	958.7 ± 144.1	890.5 ± 143.1
P wave 0 hr	119.5 ± 22.5	115.5 ± 17.8	115.6 ± 17.8	125.0 ± 27.6
PR interval 0 hr	173.6 ± 28.0	169.29 ± 28.7	172.7 ± 29.8	177.7 ± 27.4
QRS duration 0 hr	91.4 ± 7.1	90.7 ± 6.7	90.2 ±7.5	92.6 ± 7.3
QT 0 hr	362.3 ± 26.0	363.3 ± 25.8	370.2 ± 27.8	357.2 ± 25.4
QTc 0 hr	383.0 ± 20.8	384.8 ± 21.7	381.4 ± 22.3	382.4 ± 20.1

* P < 0.05 for halofantrine group versus halofantrine and kolanut

The plasma levels of potassium, sodium, creatinine, and the liver enzymes in the three groups were within the reference intervals at baseline, Table 1b.

**Table 1b:** Baseline biochemical parameters of healthy volunteers administered halofantrine alone or with either fluconazole or kolanut

Biochemical Parameters	All Subjects N = 8	Halofantrine n = 2	Halofantrine with kolanut n = 4	Halofantrine with fluconazole n = 2
Alanine transaminases (ALT)	8.3 ± 6.3	8.5 ± 4.9	4.8 ± 2.2	15.0 ± 9.9
Potassium	3.85 ± 0.2	3.95 ± 0.1	3.75 ± 0.3	3.95 ± 0.1
Sodium	139.3 ± 4.6	142.0 ± 7.1	137.5 ± 4.2	140.0 ± 4.2
Urea	21.5 ± 2.6	21.5 ± 0.7	22.3 ± 3.3	20.0 ± 2.8
Creatinine	0.88 ± 0.26	1.15 ± 0.21	0.83 ± 0.3	0.70 ± 0.0

Baseline electrocardiographic intervals significantly correlated with 6-hour values.

**Figure 1:**
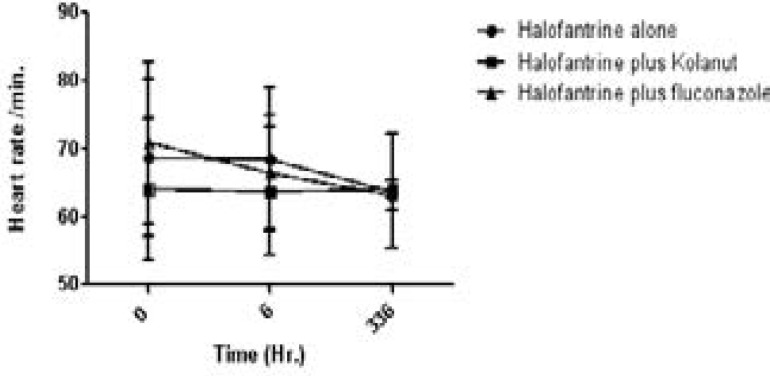
Heart rate (/min.) of study participants on halofantrine alone or in combination with kolanut or fluconazole

**Figure 2:**
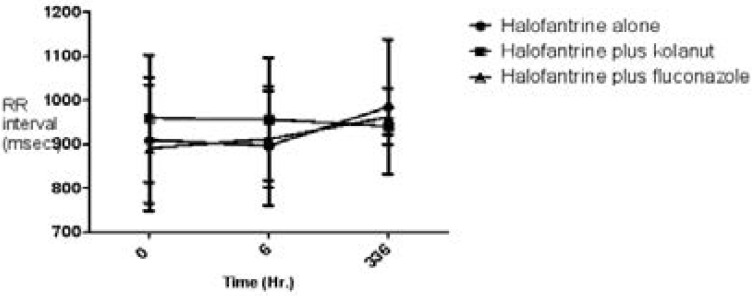
RR interval (msec) of study participants on halofantrine alone or in combination with kolanut or fluconazole

Heart rates (beats/min) and RR interval (msecs) are shown on Figures 1 and 2.

**Figure 3:**
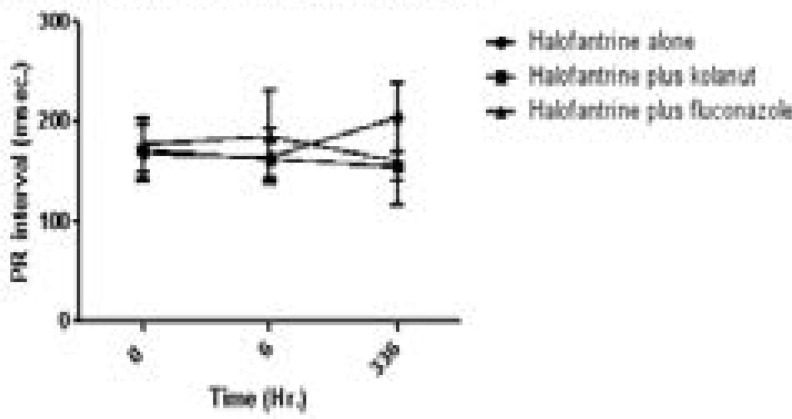
PR interval (msec) of study participants on halofantrine alone or in combination with kolanut or fluconazole

Eight individuals, halofantrine group (2), halofantrine and kolanut group (1), and halofantrine and fluconazole group (5), had pre-treatment PR interval more than 200 msec though they had no clinical symptoms; 5 individuals had PR interval prolonged at 6-hour, same number in the halofantrine and halofantrine kolanut group, but less number, 3 in the halofantrine plus fluconazole group. PR interval, Figure 3, were shortened by halofantrine alone and halofantrine with kolanut (169.29 28.67 to 165.29 28.007 and 172.73 29.843 to 163.00 18.336 msec) but was prolonged by halofantrine with fluconazole (177.70 27.394 to 186.59 44.434 msec).

**Figure 4:**
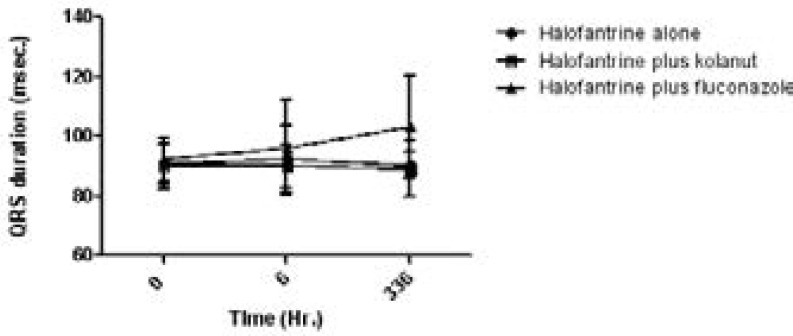
QRS duration (msec) of study participants on halofantrine alone or in combination with kolanut or fluconazole

**Figure 5:**
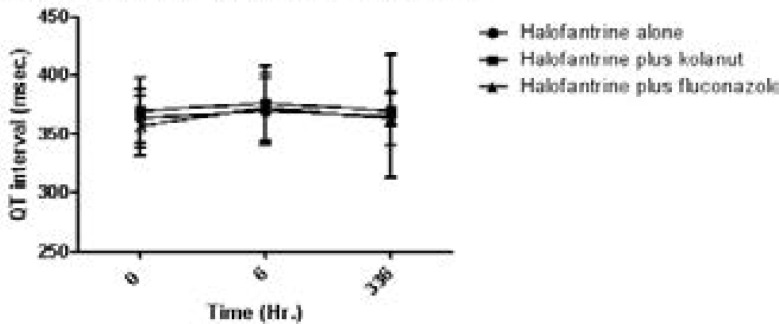
QT interval (msec) of study participants on halofantrine alone or in combination with kolanut or fluconazole

The QRS duration (msec) and QT interval (msec) are shown on Figures 4 and 5 respectively.

**Figure 6:**
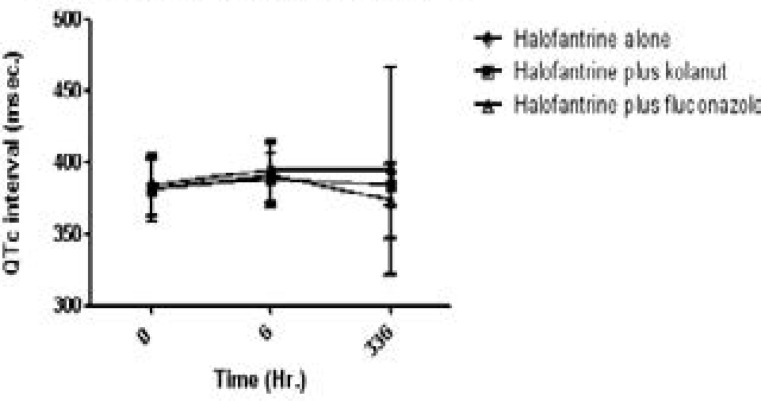
QTc interval (msec) of study participants on halofantrine alone or in combination with kolanut or fluconazole

QTc was derived from Bazzett's formula QT/√RR. There was prolongation of QTc (384.76 21.727 to 394.12 21.525; 381.36 22.29 to 388.30 17.26 and 382.35 20.08 to 390.84 21.97 msec) in all the three treatment groups at 6 hours but were not statistically significant, Figure 6.

Though no patient had QTc prolongation greater than 440 msec pre-treatment, it occurred in one patient on halofantrine and fluconazole at 6 hours, and in another on Day 14. Pre-treatment PR interval (PR _0_) correlated well with post-treatment intervals PR _6_, and with PR _14_ r= 0.519, p= 0.014; r=0.664, p=0.013. QT and QTc at 6 hrs and Day 14 correlated well with pre-treatment value. Concomitant intake of kolanut with halofantrine was reported to significantly decrease Cmax and AUC of both halofantrine and the metabolite desbutylhalofantrine. The pharmacokinetic profile observed from the plasma concentrations of halofantrine and the active metabolite desbutylhalofantrine, though appeared consistent with the PR interval reduction was not consistent with the QTc prolongation.

## Discussions

In this study, baseline electrocardiographic intervals significantly correlated with 6-hour values. QT and QTc at 6 hrs and 336 hrs (Day 14) correlated well with pre-treatment value. Electrocardiographic monitoring at 6 hours may be a most important single point measurement of assessment of the electrocardiographic changes in the heart following use of halofantrine. Halofantrine, a phenantherene methanol has a half-life of 4 days and is metabolized to an active metabolite, N-desbutylhalofantrine by cytochrome P 450 (CYP3A4). Subjects, who were healthy volunteers, were not on any of the drugs known to be associated with prolonged QT interval and the serum potassium levels were within normal limits.

Concomitant intake of kolanut with halofantrine was found to significantly decrease Cmax and AUC of both halofantrine and the metabolite desbutylhalofantrine^10^. Co-administration of fluconazole with halofantrine did not significantly alter the pharmacokinetics of halofantrine, except for the prolonged elimination half-life, however fluconazole reduced the plasma concentrations (Cmax, AUC, and metabolite ratio) but increased the Tmax of the active metabolites^11^. The pharmacokinetic profile observed from the plasma concentrations of halofantrine and the active metabolite desbutylhalofantrine, though appeared consistent with the PR interval reduction was not consistent with the QTc prolongation. Quinolones and related antimalarial drugs may have class 1c drug properties, delay ventricular repolarization and cause wide QRS complexes, however, halofantrine and quinine exhibit class 3 drug properties, cause significant QT prolongation^19^ and parenteral administration may cause significant hypotension. Halofantrine rather than the metabolite, N-debutyl-halofantrine correlated better with QTc prolongation^20^; QTc interval prolongation increased from 17 +/- 6 ms to 31 +/- 12 ms for halofantrine alone and halofantrine with grape juice respectively because grape juice inhibiting CYP3A4, inhibited the metabolism of halofantrine. The effect on QTc by halofantrine is dependent on the exposure^15^, QT/QTc increased with each dose of halofantrine 500mg administered every 6 hours for 3 doses, reaching a maximum between 18 -24 hours before returning to the baseline levels^21^. We recorded reduced PR interval which appears consistent with the observed pharmacokinetic interaction between halofantrine and kolanut or fluconazole of reduction in plasma concentration of halofantrine and the active metabolite, desbutylhalofantrine^10^,^11^, Sowumi et al reported in children an abnormally prolonged PR with halofantrine, but a similarly prolonged QTc when compared to chloroquine chlorpheniramine administration^22^,^23^, electrocardiographic abnormalities did not produce rhythm disturbance or any clinical symptoms. In children treated with halofantrine 8mg/kg 6 hrly for 3 doses, PR interval was significantly prolonged, 1^st^ degree atrioventricular (AV) block and second-degree AV block were observed at 8h, or 8 and 48 hour and at 48hours. QTc prolongation was observed up till 72 hours, but no significant changes by 168 or 336 hours23. Female gender and advancing age in elderly are risk factors for prolonged QT interval^24^. Our study participants were young males.

## Conclusion

In conclusion we found halofantrine alone or in concomitant use with fluconazole caused prolonged QT interval which correlated with the baseline, whereas use of kolanut with halofantrine caused reduced PR interval. Further studies are however needed to determine if such QT prolongation is accentuated by presence of background hitherto undiagnosed abnormality or an accentuation of such abnormality.
